# High tie versus low tie of the inferior mesenteric artery: a protocol for a systematic review

**DOI:** 10.1186/1477-7819-9-147

**Published:** 2011-11-09

**Authors:** Roberto Cirocchi, Eriberto Farinella, Stefano Trastulli, Jacopo Desiderio, Giorgio Di Rocco, Piero Covarelli, Alberto Santoro, Giammario Giustozzi, Adriano Redler, Nicola Avenia, Antonio Rulli, Giuseppe Noya, Carlo Boselli

**Affiliations:** 1 Department of General Surgery, University of Perugia, St. Maria Hospital, Terni, 05100, Italy; 2 Department of Surgical Sciences, Sapienza University of Rome, Rome, Italy; 3 Department of General and Oncologic Surgery, University of Perugia, Perugia, Italy

## Abstract

In anterior resection of rectum, the section level of inferior mesenteric artery is still subject of controversy between the advocates of high and low tie. The low tie is the division and ligation to the branching of the left colic artery and the high tie is the division and ligation at its origin at the aorta. We intend to assess current scientific evidence in literature and to establish the differences comparing technique, anatomy and physiology. The aim of this protocol is to achieve a meta-analysis that tests safety and feasibility of the two procedures with several types of outcome measures.

## Background

Nowadays surgery for rectal cancer (anterior resection or abdomino-perineal amputation) has been well standardized both ways in open and laparoscopic approach [1].

**Figure 1:**
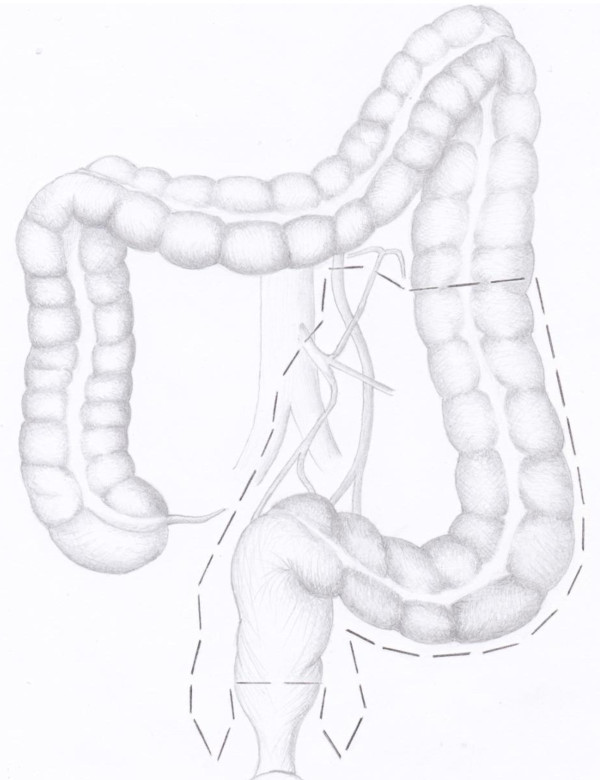
**evaluation of colon and blood supply before high tie of the inferior mesenteric artery in anterior resection of the rectum**. The image shows the level and type of vascular ligation to perform.

**Figure 2:**
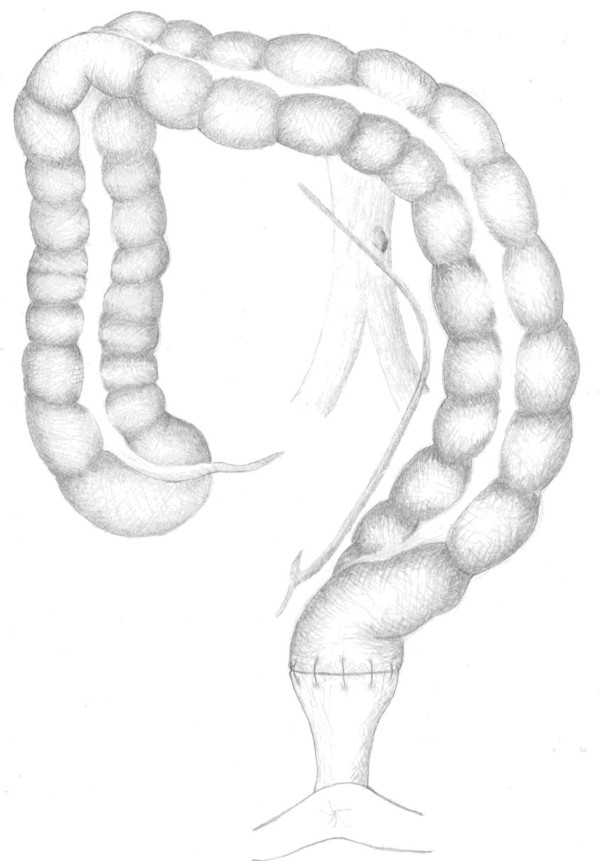
**colon and blood supply after high tie of the inferior mesenteric artery**.

**Figure 3:**
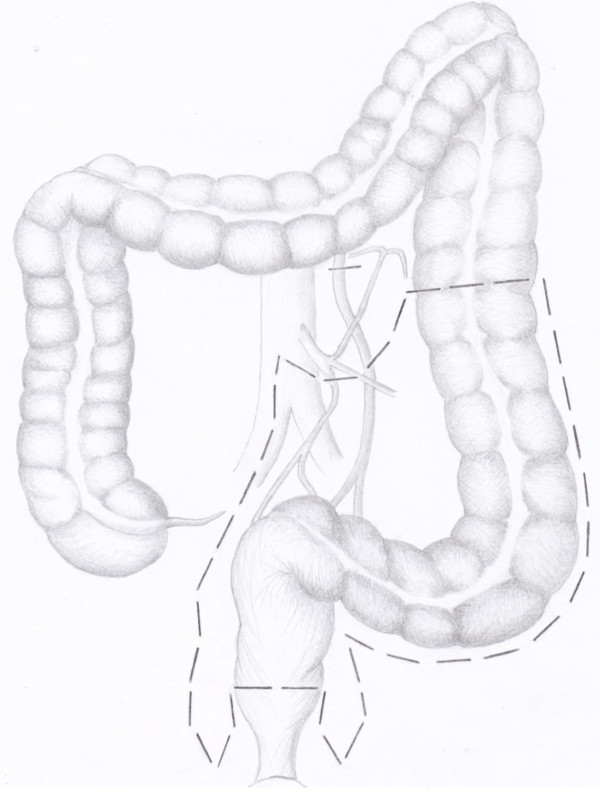
**colon and blood supply before low tie of the inferior mesenteric artery in anterior resection of the rectum**. It's shown the mode to perform this procedure.

**Figure 4:**
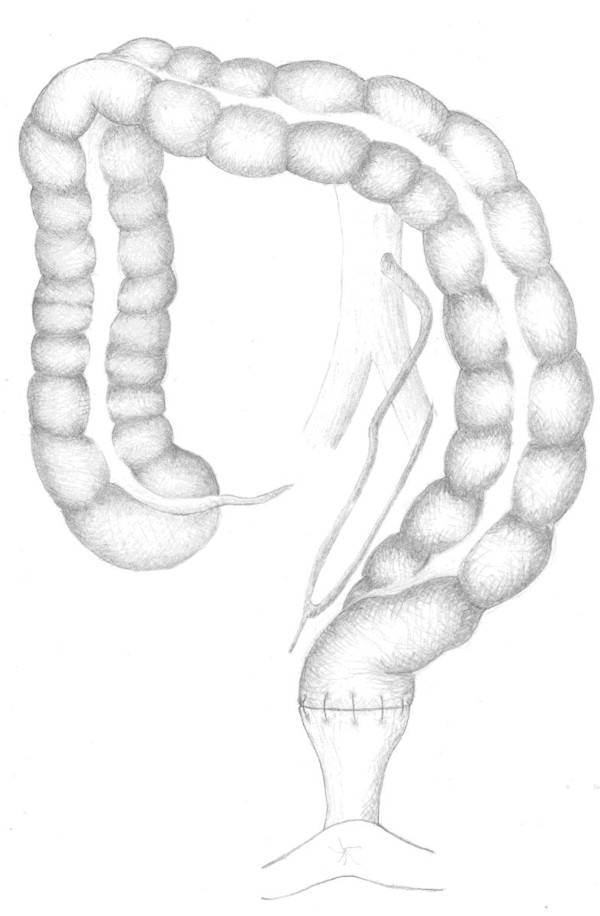
**colon and blood supply after low tie of the inferior mesenteric artery**.

In point of the fact, there are still disputes regarding the level how to execute the section of the inferior mesenteric artery (IMA): the origin from the aorta (high tie, Figure 1 and 2) or below the origin of the left colic artery (low tie, Figure 3 and 4) [2]. The alternative to the section of the IMA is its preservation, adduced by Valdoni [3]; this technique has been abdicated by most surgeons because it does not seem to assure a radical surgery for cancer. In 1959 Dunphy suggested a modified procedure instead of high ligation, in which fatty tissues and nodes were dissected free and excised in the angle between the IMA and aorta, and the artery was ligated below the left colic artery; this technique represented a compromise between the high and low ligation [4].

**Figure 5:**
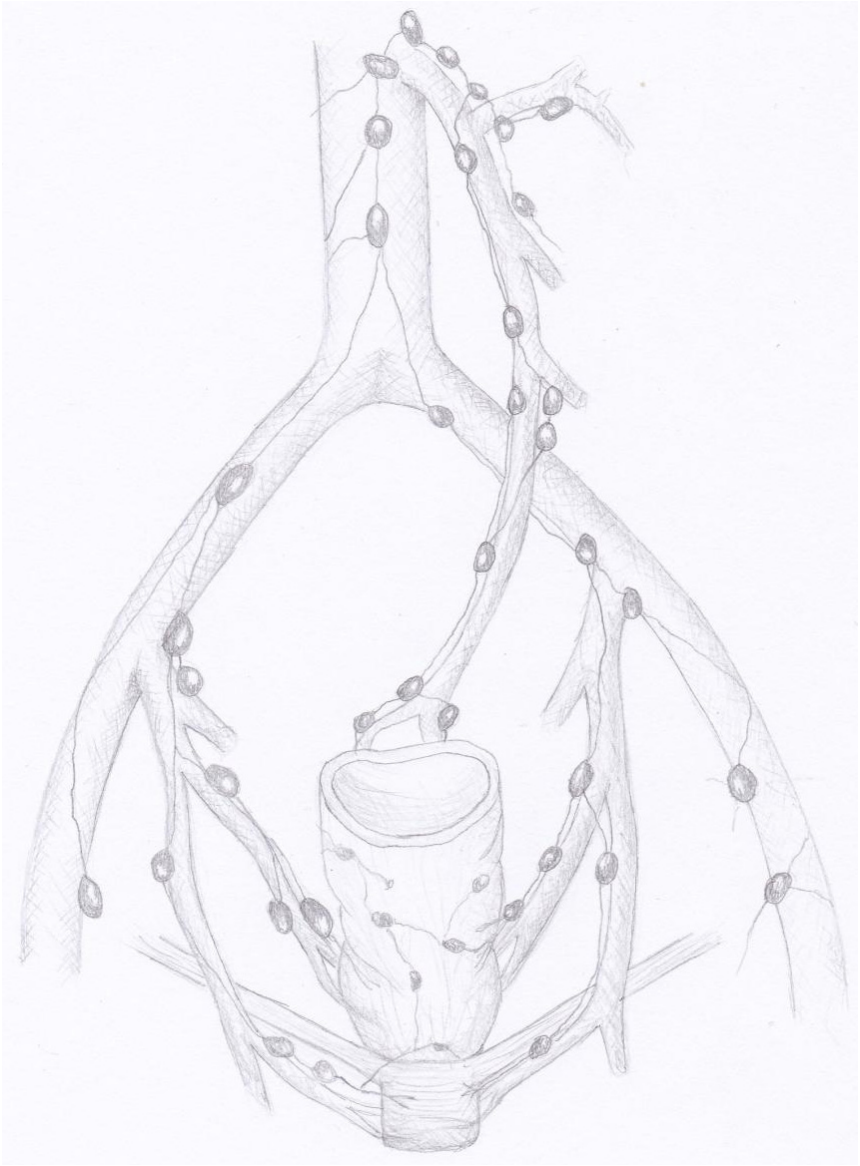
**the image shows the direction of lymphatic drainage of lower, middle and upper rectum and allows to identify the location of high and low tie areas along the inferior mesenteric artery**.

Over the years, we have seen a ceaseless debate between surgeons favorable to the low [5,6] or the high tie (Figure 5) [7,8].

Nowadays the spread of laparoscopy has encouraged more frequent execution of the high tie, which appears easier to achieve than the low tie [9-13].

The high tie also has the advantage of a lower anastomosis traction [14,15] and the disadvantage of the worst vascularization of the stumps [16-18].

Neither of these techniques assures to be superior to another, this is the opinion of two experts and of two literature reviews [19,20]. Recently a systematic review of the literature has displayed a significant advantage to accomplish the high tie [21].

## Objectives

The aim of our systematic review is to appraise the real advantages of the high and low tie of the IMA.

## Materials and methods

All aspects of the Preferred Reporting Items for Systematic Reviews and Meta-analyses (PRISMA) statement will be followed.

## Eligibility Criteria

### Inclusion criteria

We will consider both ways, randomized and non-randomized studies which compare high tie (ligation at the aortic origin) versus low tie (ligation below the origin of the left colic artery) of the IMA for sigmoid or rectal resection for cancer. Furthermore, in order to be considered for inclusion, studies have to report outcomes for sigmoid (left colectomy) or rectal cancer surgery (anterior resection/sphincter-sparing surgery or abdomino - perineal resection) and to compare high tie versus low tie. We will not impose any language or publication status restrictions.

### Exclusion criteria for study

The studies will be excluded from analysis if the outcomes of interest will not report the two techniques or whether it will be not possible to extrapolate them from the published results, also studies will be about benign lesions or inflammatory bowel disease without a distinct group of patients with cancer.

### Types of participants

Patients of any age and sex with sigmoid or rectal cancer will be considered.

### Types of surgery

Both sigmoid or rectal resection with high and low IMA tie.

### Types of outcome measures

The following outcomes will be observed:

#### Primary outcomes

Postoperative morbidity

Overall colonic cancer at 5 year survival rate

Overall rectal cancer at 5 year survival rate

#### Secondary outcomes

Postoperative mortality

Anastomotic leakage

Disease free survival colonic cancer at 5 year survival rate

Disease free survival rectal cancer at 5 year survival rate

### Information sources and search

A systematic search will be conducted in: Medline, Embase, Cochrane Central Register of Controlled Trials, CINAHL, BioMed Central, Science Citation Index and performed on all studies for potentially relevant trials comparing high with low IMA tie. A secondary search will be conducted reviewing unpublished literature databases including: Greynet, SIGLE, National Technological Information Service, British Library Integrated catalogue, Current Controlled Trials and the Cochrane Central Register of Controlled Trials

Combinations of the following search terms will be used: inferior mesenteric artery'; 'lymph node' or 'lymph nodes'; 'colon' or 'rectum'; 'cancer', 'neoplasia', 'tumour', or 'tumor'.

We will search the related article of Pub Med and all references.

To minimize retrieval bias we will perform a new manual search method that utilize the Google Scholar database and manually searched seven high-impact journals, chosen on the basis of the frequency of articles and on expert opinion.

The reference lists of all potentially eligible studies will be reviewed. Researchers who may have carried out relevant studies will be contacted. Animal trials will be excluded.

### Study Selection

Two authors (RC and CB) will assess titles or abstracts of all identified studies independently and exclude all the irrelevant ones. Full text articles of potentially relevant studies will be obtained. These studies will be assess independently in an unblended standardized manner by the two authors (GDR and AS) as to whether they met the inclusion criteria for this review.

### Data collection process

We will develop a data extraction sheet (based on the Cochrane Consumers and Communication Review Group's data extraction template), pilot tested it on ten randomly-selected included studies and refined it accordingly. One author (ST) will extract the data from the included study and the second author (GN) will check the extracted data. Disagreements will be solved through discussion, if necessary, by involving an independent third author (AR).

### Data items

The following information will be extracted by one author (ST) for each included trial:

- Year and language of publication.

- Country in which the trial was conducted.

- Year of conduct of trial.

- Single-center or multicenter trial.

- Characteristics of trial participants

- Inclusion and exclusion criteria.

- All outcomes

### Statistical analysis

Two authors (ST and EF) will perform the statistical analysis in line with recommendations from the PRISMA statement [22] and the Cochrane Handbook for systematic reviews [23]. Statistical analysis for categorical variables will be performed by using the odds ratio (OR) as summary statistic. This ratio represents the odds of an adverse event occurring in the high tie group compared with the low tie. The Mantel-Haenszel method will be used to combine the ORS for the outcomes of interest) [24,25]. For continuous variables statistical analysis, we will use the weighted mean difference (WMD). A negative WMD favored the high tie group and subgroups, and the estimated point of the WMD will be considered statistically significant with P < 0.05, if 95% confidence interval (CI) did not include the value zero.

Fixed effect models and Random effect model will be initially calculated for all outcomes. Then we will test the homogeneity among the studies by calculating the chi^2 ^and I^2^. I^2 ^or "inconsistency" describes the proportion of total variation in studies and it is independent from the number of combined studies. If the test rejects the assumption of homogeneity of the studies, then it is not appropriate to use a fixed effect model, and random effect analysis will be reported. Sensitivity analyses will also lead to explore the statistical heterogeneity [26].

If the studies report their continuous variables as medians with ranges that a meta-analysis cannot use, we will assume the mean to be equal to the median value itself and estimated the standard deviation (SD) as a quarter of the range (samples = 70) or one sixth of the range (samples > 70) [27]. If neither range nor other measure of dispersion will be reported, it will be impossible to estimate the mean and the SD based on the published data and the corresponding continuous variables will be excluded from the statistical pool.

Statistical analysis will be conducted by using the statistical software Review Manager Version 5.0.

### Assessment of quality and bias risk of the included studies

Two authors will assess the risk of bias of the trials independently (ST, JD) using for the RCTs the instructions given in the Cochrane Handbook for Systematic Reviews of Interventions and for CCTs the modified Newcastle-Ottawa scale [28,29]. Graphical exploration with funnel plots will be used to evaluate publication bias [30].

### Strategy for data synthesis

A narrative synthesis of the included studies, risk of bias and results will be performed. If heterogeneity will present a I^2 ^< 50% will reported the outcome results using a random effects meta-analysis. We will conduct sensitivity analyses based on study quality.

### Dissemination plans

The article will be submitted to a peer-reviewed journal.

### Organizational affiliation of the review

University of Perugia

### Anticipated or actual start date

1 September 2011

### Anticipated completion date

1 December 2011

## Competing interests

The Authors state that none of the authors involved in the manuscript preparation has any conflicts of interest towards the manuscript itself, neither financial nor moral conflicts. Besides none of the authors received support in the form of grants, equipment, and/or pharmaceutical items.

## Authors' contributions

Each author has participated sufficiently to take public responsibility for appropriate portions of the content. All authors contributed equally to this work, read and approved the final manuscript.
